# Syllable-Initial Phonemes Affect Neural Entrainment to Consonant-Vowel Syllables

**DOI:** 10.3389/fnins.2022.826105

**Published:** 2022-06-14

**Authors:** M. Oana Cucu, Nina Kazanina, Conor Houghton

**Affiliations:** ^1^Department of Computer Science, University of Bristol, Bristol, United Kingdom; ^2^School of Psychological Sciences, University of Bristol, Bristol, United Kingdom; ^3^International Laboratory of Social Neurobiology, Institute for Cognitive Neuroscience, National Research University Higher School of Economics, HSE University, Moscow, Russia

**Keywords:** electroencephalography, speech, syllable, neurolinguistics, entrainment

## Abstract

Neural entrainment to speech appears to rely on syllabic features, especially those pertaining to the acoustic envelope of the stimuli. It has been proposed that the neural tracking of speech depends on the phoneme features. In the present electroencephalography experiment, we examined data from 25 participants to investigate neural entrainment to near-isochronous stimuli comprising syllables beginning with different phonemes. We measured the inter-trial phase coherence of neural responses to these stimuli and assessed the relationship between this coherence and acoustic properties of the stimuli designed to quantify their “edginess.” We found that entrainment was different across different classes of the syllable-initial phoneme and that entrainment depended on the amount of “edge” in the sound envelope. In particular, the best edge marker and predictor of entrainment was the latency of the maximum derivative of each syllable.

## 1. Introduction

Electroencephalography (EEG) and magnetoencephalography (MEG) experiments show that neural phase locking to the low frequency components of the speech envelope, usually 4–8 Hz, corresponding to the theta band, is important for speech processing and comprehension (Luo and Poeppel, 2007; Peelle and Davis, 2012; Ding et al., 2017b; Khalighinejad et al., 2017). It is thought that this syllabic rhythm acts as a temporal window for speech chunking, thus contributing to the successful tracking of the acoustic signal (Arnal and Giraud, 2012; Giraud and Poeppel, 2012).

The mechanism supporting neural entrainment is still undecided; it could occur as a result of phase resetting of endogenous neural oscillations to the syllabic rhythm (Schroeder and Lakatos, 2009), via evoked neural activity triggered by the acoustic edges of the syllables (Kojima et al., 2021), or as a combination of these two (Obleser and Kayser, 2019). So far, it remains unclear what “acoustic edges” specifically represent. It is thought that these may be the high-frequency amplitude modulations of the the envelope (Zoefel and VanRullen, 2015): these may be related to the phonemic content of speech (Tallal, 2004) but could also be influenced by a number of other factors such as stress and sound intensity (Goswami and Leong, 2013). Nonetheless, it has been shown that acoustic landmarks reliably influence the amount of neural theta phase locking to speech.

Using degraded sound stimuli, which were denuded of high-frequency information and with a variety of envelope manipulations, Doelling et al. (2014) showed that the amount of phase coherence between theta oscillations and speech was correlated with the “sharpness” of the stimuli, measured as the mean sum of the positive derivative of the acoustic envelope. Similarly, it was found in Heil (1997) and Heil (2003) that the spiking activity of auditory neurons showed time-sensitive responses to the derivative of the envelope of sound stimuli: the response times and precision of the spikes, measured in the primary auditory cortex of cat, were dependent on the fluctuations in the derivative of the sound envelope at its onset. Moreover, an MEG study demonstrated that the latencies and amplitudes of the P50 and N100 auditory responses varied with the rise times of sound envelopes (Biermann and Heil, 2000).

There is debate as to where in a syllable the most important features for neural entrainment are to be found. It has been suggested, for example, that speech chunking is aided by the acoustic information located at syllabic onsets. The idea of syllabic onsets acting as landmarks for entrainment has been since supported by Zoefel and VanRullen (2015). More recently, in an electrocorticography (ECoG) experiment which measured responses to natural speech, Oganian and Chang (2019) found that the correlations between neural activity and the speech envelope were highest at the times of the maximum derivative of the envelope of each syllable; these landmarks are thought to convey the maximum rate of change in the envelope and are thus likely to correspond to formant or consonant-vowel (CV) transitions. An alternative view suggests that the syllabic or vowel peaks, closer to the middle of the syllable, are the most important landmarks for phase locking to speech because they carry the highest amount of acoustic energy to which neural oscillations subsequently align (Ghitza, 2013).

Our experiment used EEG to measure the neural entrainment to different phonemes; the amount of entrainment was quantified by calculating inter-trial phase coherence (ITPC), a commonly used measure of neural entrainment to a periodic or quasi-periodic stimulus. We investigated whether CV syllables beginning with different consonants, as well as vowel-only syllables, were different in the quality of acoustic edges they provided, and whether listening to streams of these syllables led to differences in neural entrainment. To ensure a robust and quantifiable entrainment we used a near-isochronous signal; this resulted in a peak in the ITPC at the stimulus frequency. We examined the relationship between voicing, place and manner of articulation of the syllable-initial consonants of the stimuli and the amplitude of the ITPC at the stimulus frequency and its harmonics. We also investigated a set of edge markers which quantify numerically edge-like properties of the acoustic envelope. We related these to the voicing and articulation of the syllable-initial consonants and calculated the relationship between the edge-markers and the ITPC peak amplitudes.

The syllables in each of our CV stimulus streams all started with the same consonant, ranging from plosives to fricatives to sonorants. The consonants were followed by one of five English vowels ([ɑ], [ɛ], [i], [ɒ], [u]) for all streams. Edge markers such as sharpness (Doelling et al., 2014), the peak envelope and maximum derivative were analyzed for each syllable, and their correlations with neural responses were calculated. We predicted that differences such as voicing and the manner of articulation would reflect in differences in neural responses: this was observed and we found a very clear relationship between the strength of neural entrainment and edge markers, such as the latency of the maximum derivative of the envelope, or the sharpness of the syllabic onset.

## 2. Methods

### 2.1. Participants

Thirty-three right-handed native English speakers, without any self-reported learning disabilities or hearing impairments, were recruited using the University of Bristol Experimental Hours System or through social media advertisements. They were rewarded for their time with either course credit or financial compensation (£10/h). Participants gave informed consent and were allowed to withdraw at any time from the study, in conformity with the University of Bristol Human Participants Ethics Guidelines. Eight participants performed poorly on the experimental task (see Procedure for further task-dependent exclusion criteria) and their data were removed from the analyses. Twenty-five participants (17 females, mean age = 23.68 years old, standard deviation = 5.28 years) were included in the final analysis.

### 2.2. Stimuli

The experimental design was within-subjects and there were 15 conditions, corresponding to the initial syllable segment. Stimuli in 14 of the conditions comprised CV syllables, and one condition contained vowel-only (V) stimuli.

The syllable stimuli were recorded from a female native English speaker in a soundproof room, using Cool Edit Pro 2.0 software (Adobe Systems Inc.). Each syllable was recorded ten times, and the clearest exemplar of each syllable was selected for the experiment. The syllables were then normalized to 70 dB sound pressure level and shortened using a custom Python script which applied the Pitch Synchronous Overlap and Add algorithm (Moulines and Charpentier, 1990) for duration modification. The target shortening duration was chosen to be 250 ms, in order to match the average speech rate of 4 Hz (Ding et al., 2017b), but the lengths of the syllables differed between each other (mean duration: 249.05 ms; standard deviation = 2.42 ms; minimum = 241.63 ms; maximum = 255.28 ms). Syllable duration differences were a consequence of using gammatone filters to preserve the original pitch of the sound; it was hoped that a slight difference in the duration of the syllables reduced the possible effects of neural adaptation to repetitive stimuli which could potentially affect entrainment. A histogram of the lengths of the syllable durations is given in Supplementary Figure 1. While the use of the gammatone filters did alter the shape of the envelope, this change was very mild and the quality of the acoustic content was not affected; the syllables sounded natural and were easily understood. Furthermore, Supplementary Figure 2 shows that the mean power of all stimulus envelopes shows peaks at the rate of 4 Hz and harmonics.

Each of the CV conditions contained one of 14 consonants. We built three separate streams for each condition, in which the order of the five vowels was pseudo-randomized so that the same vowel was not repeated in consecutive syllables. For example, in the vowel-only condition, the first five syllables of each stream were, in order:

**Table:** 

/a/	/u/	/e/	/a/	/i/	…
/e/	/a/	/i/	/u/	/e/	…
/i/	/o/	/u/	/o/	/a/	…

The order of the vowels was then kept the same for the three streams of each condition. For example, the first five syllables of each stream in the /b/ condition were:

**Table:** 

/ba/	/bu/	/be/	/ba/	/bi/	…
/be/	/ba/	/bi/	/bu/	/be/	…
/bi/	/bo/	/bu/	/bo/	/ba/	…

The streams were 5 s long and contained 20 syllables. Each stream was repeated 10 times. Filler stimuli were also used to ensure participant attention throughout the experiment. Fillers were stimuli which contained a single syllable starting with a different consonant from the dominant one (for example a single /fa/ syllable in a /b/ stream, such that /ba/ /bo/ /bi/ /fa/ /be/ would be the last five syllables in the stream). Participants were required to detect the “different” syllable, which was always inserted in the second half of the stimulus. Each filler stimulus was only presented once. In total, there were 450 target stimuli, including repetitions, and 50 filler stimuli. All stimuli are freely available on the Open Science Framework website (see Data Availability).

### 2.3. Apparatus

A 32-channel Brain Products EEG cap (Brain Products GmbH, Gilching, Germany) was used to record scalp activity (see channel names and configuration in Supplementary Figure 3). Electrolyte gel (SuperVisc, EASYCAP GmbH, Herrsching, Germany) was inserted through indentations in the electrodes and onto the scalp to increase conductivity. The impedance of each electrode was kept below 5 kΩ. The stimuli were delivered using Presentation software (Neurobehavioral Systems, Inc.) through a pair of Sony Stereo headphones (model MDR-XD100, Sony Europe Ltd.) placed comfortably on the participants' heads over the EEG cap. EEG activity was recorded at 1 KHz sampling rate using actiCap equipment and BrainVision Recorder software (Brain Products GmbH, Gilching, Germany).

### 2.4. Procedure

The duration of the EEG setup was approximately 40 min, and the experiment lasted up to 65 min. After each filler stream, a question appeared on the screen asking participants to type in the “different” syllable. No question was asked after a non-filler stream. Participants typed “none” if they could not hear a different syllable. A sad or a smiling emoji was shown on the screen as feedback for performance after each keyboard response. Participants were given examples of target syllables before the experiment, with the correct spelling for each vowel so that their performance was not affected by spelling, but only by the degree of attention or intelligibility.

A practice block was played in the beginning of the experiment. This contained four /b/ stimuli, in which the orders of the vowels were different than the ones used in the main experiment, and two fillers comprising the same dominant syllable-initial consonant, which were also not present in the main experiment. Participants with more than 50% incorrect responses to filler stimuli were eliminated from the analysis.

There were five experimental blocks, which lasted approximately 12 min each and contained 100 individual streams of syllables. The stimuli were pseudo-randomized so that each block comprised ten fillers and 90 experimental streams, that is, ten repetitions of three separate streams for three different consonants. These stimuli were not played in any of the other blocks. The inter-stimulus interval was 2 s. Each 12-min block was followed by a break that was self-paced but no shorter than 30 s.

### 2.5. Data Analysis

#### 2.5.1. EEG

All EEG data were processed in Matlab (version R2018b, MathWorks, Inc.), using the EEGLAB toolbox (Delorme and Makeig, 2004) for pre-processing. The data were low-pass filtered at 50 Hz, high-pass filtered at 1 Hz, re-referenced to the average of all channels and split into 5-s epochs. We used custom scripts for time-frequency analyses which are available online (see Data Availability). We conducted ICA to remove eye-movement related components. A component was removed if the frontal channels contained more than 12% of total EEG power; this followed the approach reported in Ding et al. (2017b) and Burroughs et al. (2021).

To prevent potential interference with transient auditory onset ERPs the first 500 ms of each epoch were removed from subsequent processing (Ding et al., 2017b). A fast Fourier transform was obtained for each epoch using a Hanning taper. Subsequently the square mean resultant, referred to in this context as the ITPC:


(1) $$R(f,\text{x})={\left(\frac{\sum_{k=1}^{K}\cos\theta (f,\text{x},k)}{K}\;\right)}^{2}+{\left(\frac{\sum_{k=1}^{K}\sin\theta (f,\text{x},k)}{K}\;\right)}^{2},$$

was calculated where θ(*f*, x, *k*) is the complex phase of the Fourier transform at frequency *f*, with **x** standing in for the other indices: the electrode (*e*), the stream (*s*) and the participant (*a*) indices. The trial index is *k* so *K* = 10 is the number of trials per stream. The ITPC is a number in the range [0, 1] where zero would correspond to a no phase coherence and one to perfect coherence. The evoked power was also calculated; this is described in the Supplementary Material.

Following Burroughs et al. (2021), peaks in the average ITPC


(2) $$R(f)=E_{s,e,a}[R(f,x)]$$

with *E*_*s, e, a*_[…] denoting the average over the *s*, *e* and *a* indices, were tested for significance by applying a Mann-Whitney *U*-test to


(3) $$R(f,a)=E_{s,e}[R(f,x)]$$

at the relevant frequency to surrogate random data; the fictive data replicated the analysis used to produce *R*(*f, a*), except the 10 trials for each ITPC values were replaced with random generation of phases. This was used to produce 5,000 fictive participants and the real data were compared to that.

To test the differences in entrainment between the different phoneme conditions, we conducted univariate repeated measures analyses of variance (ANOVA), with Greenhouse-Geisser corrections where the assumption of sphericity was not met. *Post-hoc* multiple comparisons used Bonferroni corrections for paired, two-tailed *t*-tests. All statistical analyses between responses to different phonemic groups were done in RStudio (version 1.2.335, RStudio, PBC). The statistical analyses were conducted on the average of all 32 channels, as is common for frequency tagging experiments (Ding et al., 2017a; Milton et al., 2020).

For *post-hoc* correlations the results are presented without any correction for multi-correllation. Since this is a *post-hoc* analysis which uses PCA components the appropriate correction is not well-established. In the event, the main correlation result we report has a *p* < 0.001 and a correction would not affect significance.

#### 2.5.2. Stimuli

In order to study the relationship between the sound “edge” and EEG responses, we calculated a number of stimulus properties, continuous measures of the prominence of the edge which we refer to as edge measures. To do this in a way which is comparable to previously reported experiments we follow the procedure described in Doelling et al. (2014) when calculating a sound envelope. This produces an envelope which mirrors in an approximate way the output from the cochlea and hence the input to the regions of interest to us. We first applied a cochlear filter of 32 log-spaced frequency bands, spanning between 80 and 8,000 Hz, to each syllable. In each frequency band the Hilbert transform was used to calculate an analytic signal which, in turn, gave a narrowband envelope. The envelope of each syllable was obtained by summing the narrowband envelopes. This summed envelope was then filtered between 2 and 10 Hz using a zero-phase finite-impulse-response bandpass filter of the order 350 with a Kaiser window (Kaiser and Schafer, 1980).

**Figure 1:**
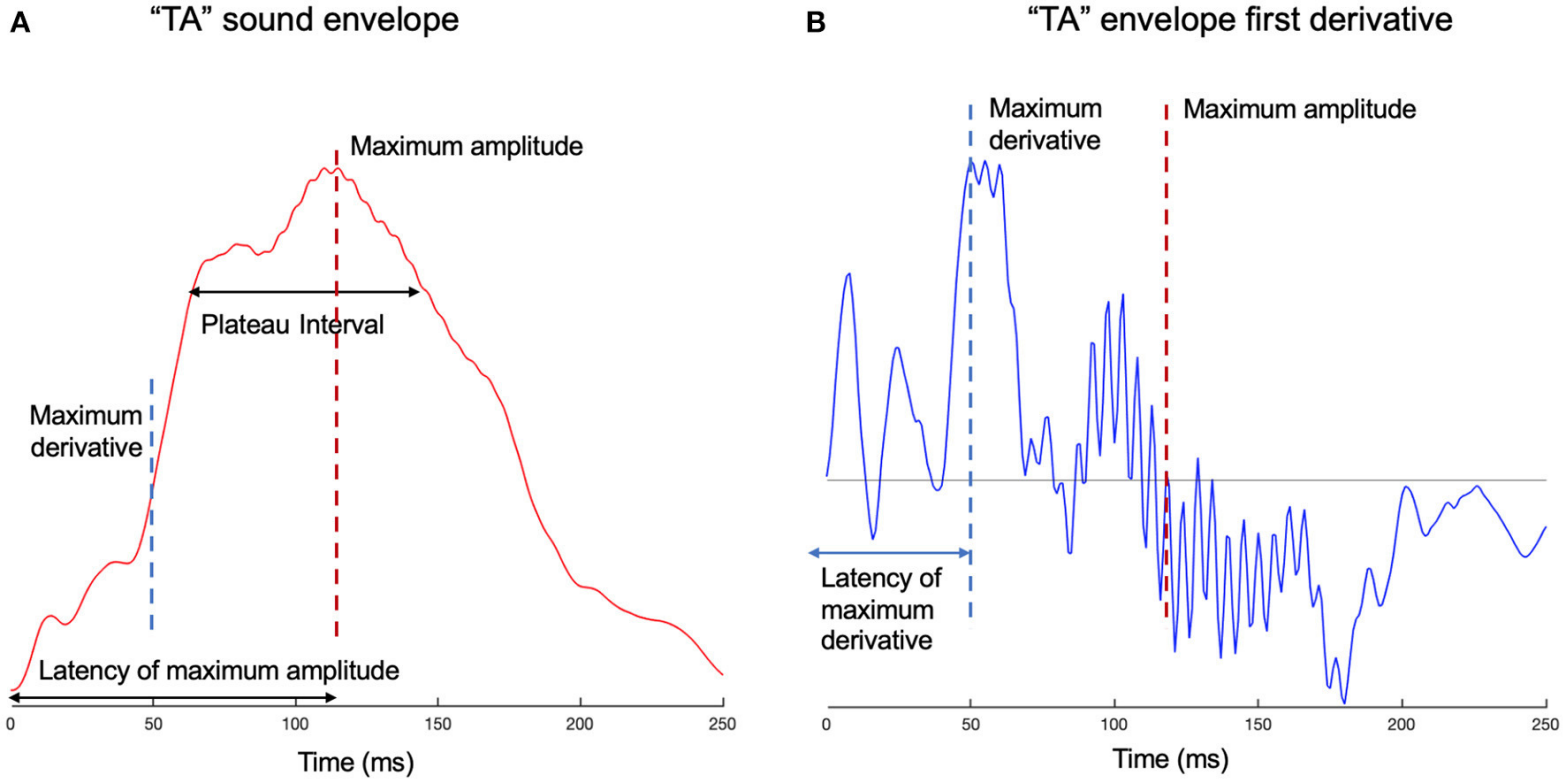
Edge measures examples for syllable “ta.” **(A)** The smooth sum of narrowband envelopes is given for syllable “ta.” The amplitude of the envelope is plotted against time. On the time axis are marked the latencies of the maximum amplitude (MA) and maximum derivative (MD), which correspond to their relevant landmarks on the amplitude axis. The plateau of the syllable peak is also shown, measured as the time from 80% of the MA on the ascending slope to 80% of the MA on the descending slope. The maximum derivative can be considered a marker for the consonant-vowel transition, which is a temporal interval, but for this particular syllable, delineates between /t/ and /a/ phonemes. **(B)** The first derivative of the envelope is shown for syllable “ta.” The maximum derivative, its latency, as well as the maximum amplitude, are shown on the graph.

We measured sharpness (Doelling et al., 2014) by taking the mean of the total positive derivative of the summed envelope. However, sharpness may not be a suitable measure of ‘edge’ for a single syllable where it is effectively a noisy measure of the maximum amplitude (MA). The MA and the latency to the MA (see Figure 1) were also measured separately. The latency to the MA is vulnerable to noise because many syllables have a plateau in their envelope with values near the MA. To avoid this we also measured the latency to a value of 80% of the MA. To measure the width of any syllable plateau we calculated the interval between the time the envelope reached 80% of MA as it increased until the point is fell back to the same value. The maximum derivative and its latency were also calculated; this followed Oganian and Chang (2019), except here the maximum derivative for the smooth sum of narrowband envelopes was used, as opposed to the broadband envelope. These measures are illustrated in Figure 1.

Lastly, we calculated the Gini index of each syllable. This is a measure, somewhat akin to Shannon's entropy, of the unevenness of a distribution, originally the distribution of income in a population (Gini, 1912). The Gini index is:


(4) $$G=\frac{\sum_{i=1}^{n}\sum_{j=1}^{n}\left|x_{i}-x_{j}\right|}{2n\sum_{i=1}^{n}x_{i}}$$

where *x*_*i*_ is the value of the envelope at the discretized time point *t*_*i*_. The Gini index reaches its maximum value of one if all but one *x*_*i*_ is zero; it has the value zero if all *x*_*i*_ are the same. Here the Gini index was used to quantify how ‘prominent’ a peak the syllable had: a prominent peak surrounded by steep ascending and descending slopes would give a high Gini index, a more uniform envelope would have a low Gini index, and, it might be considered to have less obvious landmarks to drive neural entrainment.

For each of the 15 conditions we determined the value of seven edge markers (sharpness, MA, the latency to MA, the latency to 80% of MA, the MD, the latency to MD and the Gini index) by calculating an average of the edge marker across all five syllables used in an individual condition.

## 3. Results

### 3.1. EEG

**Figure 2:**
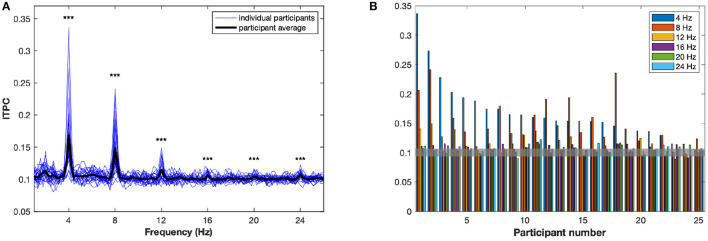
The inter-trial phase coherence (ITPC). **(A)** The ITPC, averaged over channels and conditions, is plotted as a function of frequency, between 1 and 26 Hz. The bold black line represents *R*(*f*), the average over all participants. Each of the blue lines represents a *R*(*f, a*), the average for an individual participant *a*. Significant peaks can be observed at 4, 8, 12, 16, 20, and 24 Hz. ***p < 0.001. **(B)** Significant peaks in ITPC are plotted for each participant. The value 0.1 of the ITPC represents the mean ITPC for random phases, at 95% confidence intervals: consequently, any bar above the value of 0.105 is above the significance threshold.

The ITPC averaged over all conditions and electrodes, *R*(*f*), is plotted as a function in Figure 2. There are peaks, significantly different from random at the stimulus rate, *f* = 4 Hz, and at the harmonics (*p* < 0.001). In order to assess to what extent these peaks reflect the same, or different, aspects of neuronal entrainment, we calculated the correlation coefficients between the values of *R*(*f, a*) at the *f* values corresponding to different peaks, that is at 4 Hz and harmonic values at 4, 8, 12, and 16 Hz. We found moderate positive relationships at 4 and 8 Hz (*r* = 0.54, *p* = 0.006), 4 and 12 Hz (*r* = 0.59, *p* = 0.002), 8 and 12 Hz (*r* = 0.56, *p* = 0.004) and 8 and 16 Hz (*r* = 0.58, *p* = 0.002). No significant correlations were observed between 4 and 16 Hz (*r* = 0.37, *p* n.s.), and at 12 and 16 Hz (*r* = 0.25, *p* n.s.).

In response to this complex interplay of dependence between the harmonics, principal component analysis (PCA) was conducted on the ITPC values *R*(*f, a*) at *f*= 4, 8, 12, and 16 Hz. The PCA loadings corresponding to the ITPC at each of the four measured frequencies are shown in the Supplementary Figure 5. Rather than focusing on a particular harmonic this PCA approach allowed us to select principal components for further analysis. For simplicity, further statistical tests were conducted only on the first two principal components of the ITPC; while these are not cleanly differentiated from other lower components by a “dog-leg” in the explained variance, the first two components accounted for a total of over 95% of the variance, with individual explained variances being 76.2 and 20.08%, respectively. We will refer to these as ITPC1 and ITPC2.

In fact, ITPC1 is a weighted average of the ITPC at all four frequencies, with *f* = 4 Hz receiving the highest weight, but with all four frequencies weighted positively. The choice of the first principal component, rather than, for example, the *f* = 4 Hz response or the average of the responses over the four harmonic frequencies, is not crucial to what follows; it has the advantage of picking out a privileged linear combination of the frequencies.

### 3.2. Effects of Phonemic Group

The relationship between the stimulus condition and neural responses was considered. The average values of ITPC1 and ITPC2 were compared across conditions by conducting one-way repeated measures ANOVAs. For the 15 conditions, ANOVA showed a significant main effect [ITPC1: *F*_(14, 24)_ = 7.89, *p* < 0.001; ITPC2: *F*_(14, 24)_ = 2.01, *p* < 0.05]. However, pairwise comparisons did not show any significant effects.

**Figure 3:**
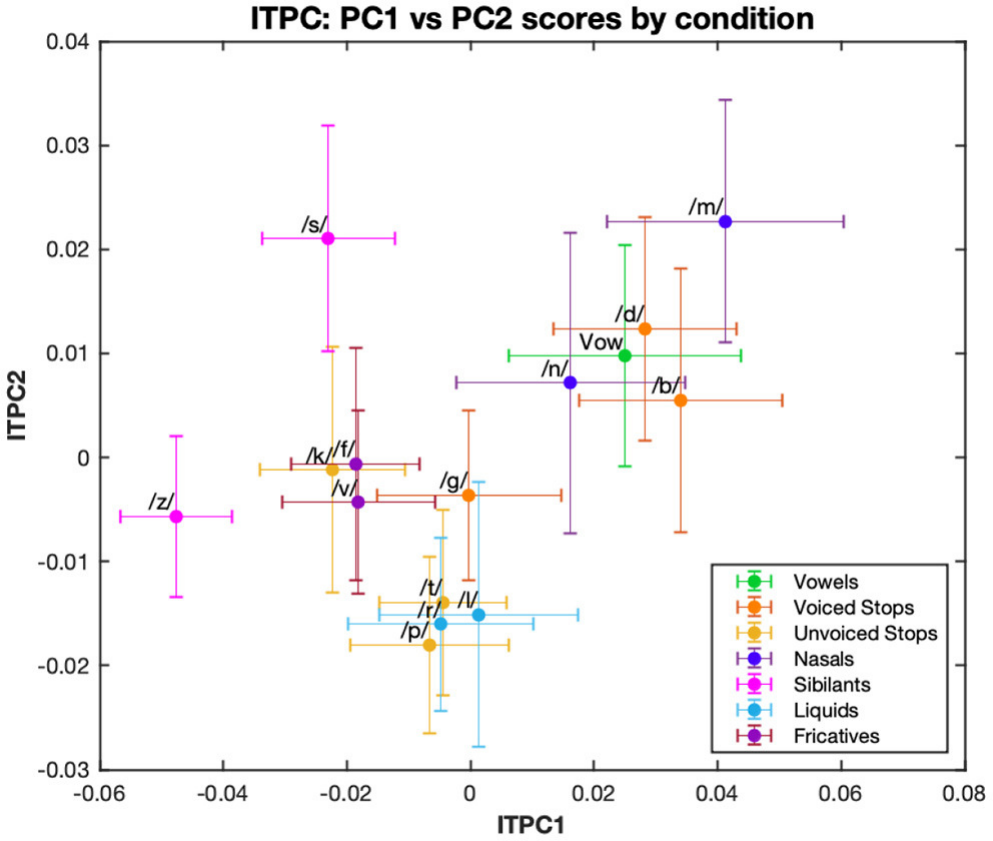
First and second principal components of ITPC. The ITPC values at 4, 8, 12, and 16 Hz are multiplied by their respective PCA loadings for the first two principal components, and then plotted for each condition. Different color graphs represent the phonemic category: vowel-only or for syllable-initial consonants, their manner of articulation.

A *k*-means cluster analysis performed on the distribution of ITPC values by condition showed three main groups: the first, containing fricative (/f/, /v/, /s/, /z/) and /k/ (an unvoiced plosive) conditions; the second, containing a mixture of liquid (/l/, /r/), unvoiced plosive (/t/, /p/) and /g/ (a voiced plosive) conditions; and finally, the group comprising nasal (/m/, /n/), voiced plosive (/b/, /d/) and vowel conditions (Figure 3). These clusters incorporate mixtures of conditions defined by phoneme features and, while this may reflect similarities across the neural processing of different manners of articulation, we were also interested in how syllable-initial consonants belonging to different linguistic classes affect neural entrainment. To investigate this more quantitatively we considered a combination into three groups, as described in Table 1. This grouping is common because of the similarity within each group in the release of the air flow during articulation (Chomsky and Halle, 1968). The vowel condition was omitted from these investigations. For ITPC1, the three groups also showed a main effect [*F*_(2, 24)_ = 12.06, *p* < 0.001]. In *post-hoc t*-tests using a Bonferroni correction for multiple comparisons, fricatives/sibilants showed significantly less phase locking than stops (*p* < 0.001) and nasals/liquids (*p* = 0.002), but there was no difference between stops and nasals/liquids.

**Table 1:** Phonemes and phoneme types used in building the stimuli.

	**Stops**	**Fricatives / sibilants**	**Nasals / liquids**	
**Consonants**				
	**b d g** k p t	f **v** / s **z**	m n / l r	
**Vowels**				
a	e	i	o	u
[ɑ]: “a” in “car”	[ɛ]: “e” in “get”	[i]: ‘ee” in “bee”	[ɒ]: “o” in “pot”	[u]: “oo” in “coo”

*There were 15 stimuli conditions depending on syllable types: 14 consonant-vowel conditions and a vowel only condition. In a subsequent analysis we considered the consonants in different groups, as listed here; we also compared voiced and unvoiced stops and fricatives/sibilants: voiced consonants are marked here in bold*.

We then considered a finer grouping into five groups separating sibilants from fricatives and nasals from liquids. For ITPC1, the ANOVA showed a main effect of group [*F*_(4, 24)_ = 11.35, *p* < 0.001]. *Post-hoc t*-tests using a Bonferroni correction for multiple comparisons revealed that entrainment was the lowest in the sibilant category, this being significantly smaller than for stops (*p* < 0.001), nasals (*p* < 0.001) and liquids (*p* = 0.007). There was also a significant difference between fricatives and nasals, with the latter eliciting higher ITPC1 values (*p* = 0.009).

**Figure 4:**
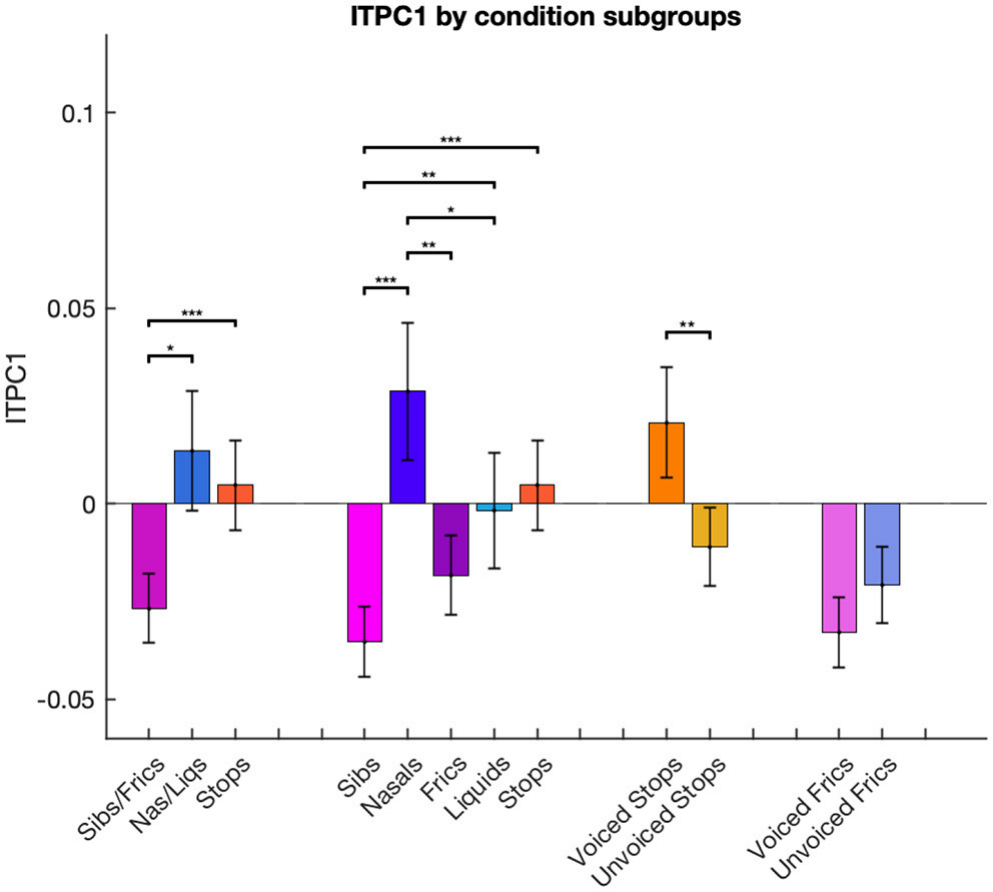
Differences in ITPC1 calculated for conditions combined in different groups. ITPC1 values in each condition are averaged across multiple phonemic subgroups: stops, fricatives/sibilants and nasals/liquids; stops, fricatives, sibilants, nasals and liquids; voiced and unvoiced stop consonants; voiced and unvoiced fricative/sibilant consonants. Error bars represent standard error of the mean. ***p < 0.001, **p < 0.01, and *p < 0.05, Bonferroni-corrected.

The effect of voicing was also examined. ITPC1 showed an opposite response to voicing in stops compared to fricatives/sibilants: voiced stops had significantly higher ITPC1 values than unvoiced ones (*p* = 0.001), while voiced fricatives/sibilants showed lower values than unvoiced ones, but this trend was only marginally significant (*p* = 0.068). These results can be seen in Figure 4.

For ITPC2, ANOVA results were significant when conducted for five [*F*_(4, 24_) = 2.73, *p* < 0.05], but not three consonant groups [*F*_(2, 24)_ = 0.24, *p*= n.s.]. *Post-hoc* Bonferroni-corrected *t*-tests conducted on the five groups indicated that nasals elicited higher entrainment than liquids (*p* = 0.008), but no other comparisons were significant.

### 3.3. Correlations Between Stimulus Edge Markers and EEG

Next, we quantified which of the edge measures affect the amount of neuronal entrainment. The correlation coefficient over the 15 stimuli conditions was calculated to compare ITPC1 and ITPC2 with each of the edge measures. The ITPC1 showed significant correlations with most of the edge measures (see Table 2), though not with the value of the MA or of the MD. There was only a marginally significant positive correlation between ITPC1 and the envelope plateau.

**Table 2:** Correlation coefficients between ITPC1 and edge markers.

**Edge markers**	***r***	***p***
Sharpness	0.6	0.018 ^*^
Maximum Amplitude (MA)	0.31	n.s.
MA Latency	–0.75	0.001 ^**^
80% of MA Latency	–0.76	<0.001 ^***^
Plateau	0.51	0.051
Gini Index	–0.61	0.016 ^*^
Maximum derivative (MD)	0.37	n.s.
MD Latency	–0.85	<0.001 ^***^

*The correlation coefficient r and its significance p where calculated in the usual way:*

* *p < 0.05,*

** *p < 0.01,*

*** *p < 0.001*.

We kept for further analyses those edge measures which led to significant or marginally significant correlations with ITPC1: these were the latencies of the MD and MA, sharpness, the Gini Index and the length of the syllable plateau. As with the ITPC, in order to provide a parsimonious description of the behavior, we applied PCA to these variables. The first two principal components explained 53.04 and 29.77% of the total variance, respectively. These two components are referred to as PC1 and PC2. The factor loadings for each component are given in the Supplementary Figure 6, indicating the contribution of each of the edge markers to each component.

**Figure 5:**
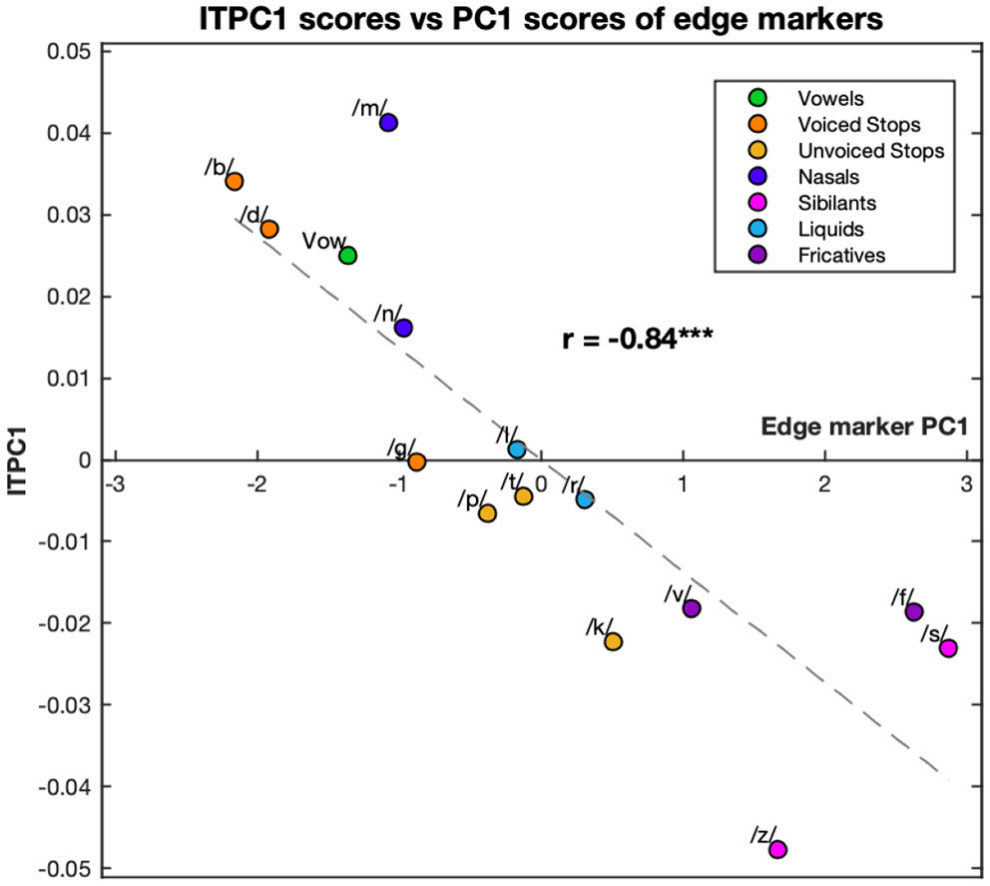
Correlation between the first principal component of the ITPC and edge markers. The values of ITPC1, corresponding to the EEG response, are plotted against the PC1 scores corresponding to different edge markers of the syllables. Both ITPC1 and PC1 values are averaged over phonemic condition. Each color corresponds to a different condition (see legend). Dashed line represents the line of best fit (least means squares). The correlation coefficient is given above this line. ***p < 0.001.

Comparing the ITPC1 and ITPC2 with PC1 and PC2 only revealed a relationship between the ITPC1 and PC1; however this relationship was very clear with *r* = −0.84 (*p* < 0.001). The relationship is graphed in Figure 5. The syllables with the highest PC1 scores led to the least entrainment, while the ones with the lowest PC1 scores led to the most phase locking.

**Figure 6:**
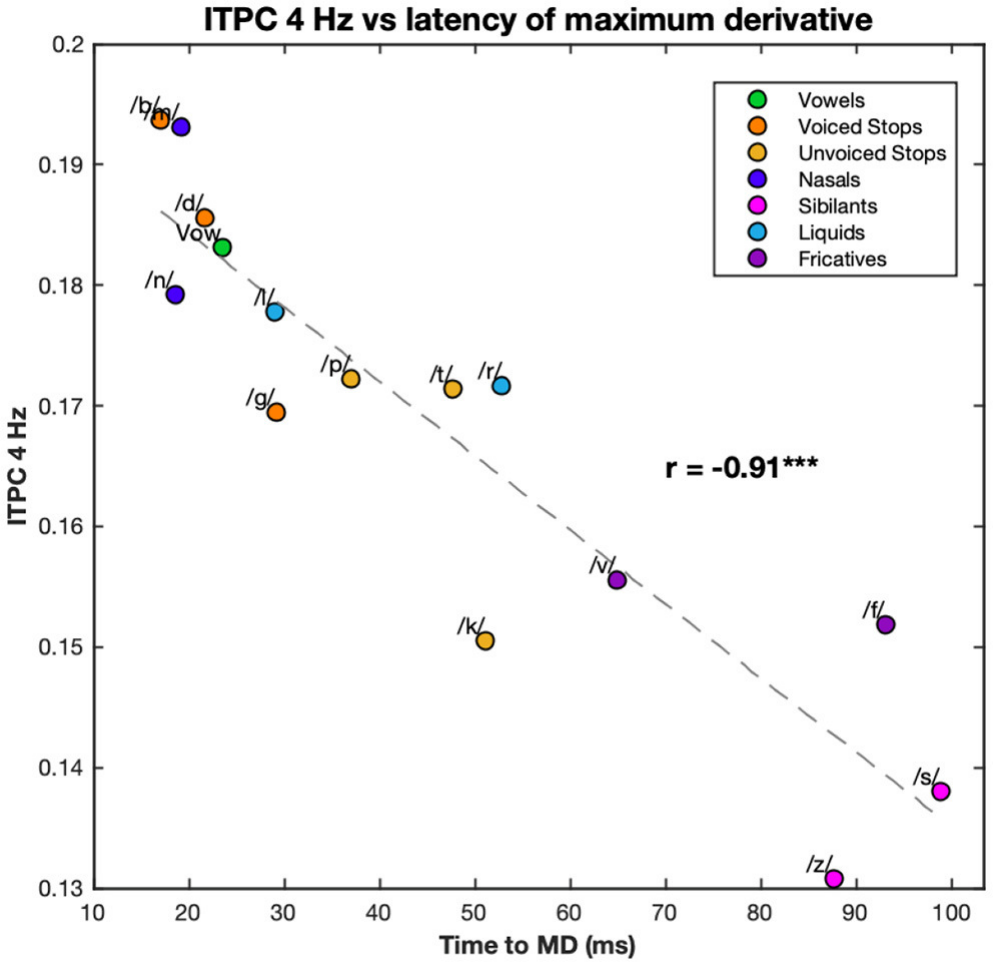
Correlation between the 4 Hz ITPC and the latency of the maximum derivative. The values of the ITPC at 4 Hz are plotted against the latencies of the maximum derivative of each syllable. ITPC and maximum derivative values are averaged according to phonemic condition. Each color corresponds to a different condition (see legend). Dashed line represents the line of best fit (least means squares). The correlation coefficient is given above this line. ***p < 0.001.

Because of the large number of harmonics and the large number of edge markers, it has proved convenient to examine the relationship between principal components. However, as the ITPC at 4 Hz was the biggest contributor to ITPC1 and the latency of MD was the biggest contributor to PC1, the relationship between these two was also calculated. They showed a clear linear relationship with correlation coefficient *r* = −0.91 (*p* < 0.001) with a similar distribution of conditions (see Figure 6).

**Figure 7:**
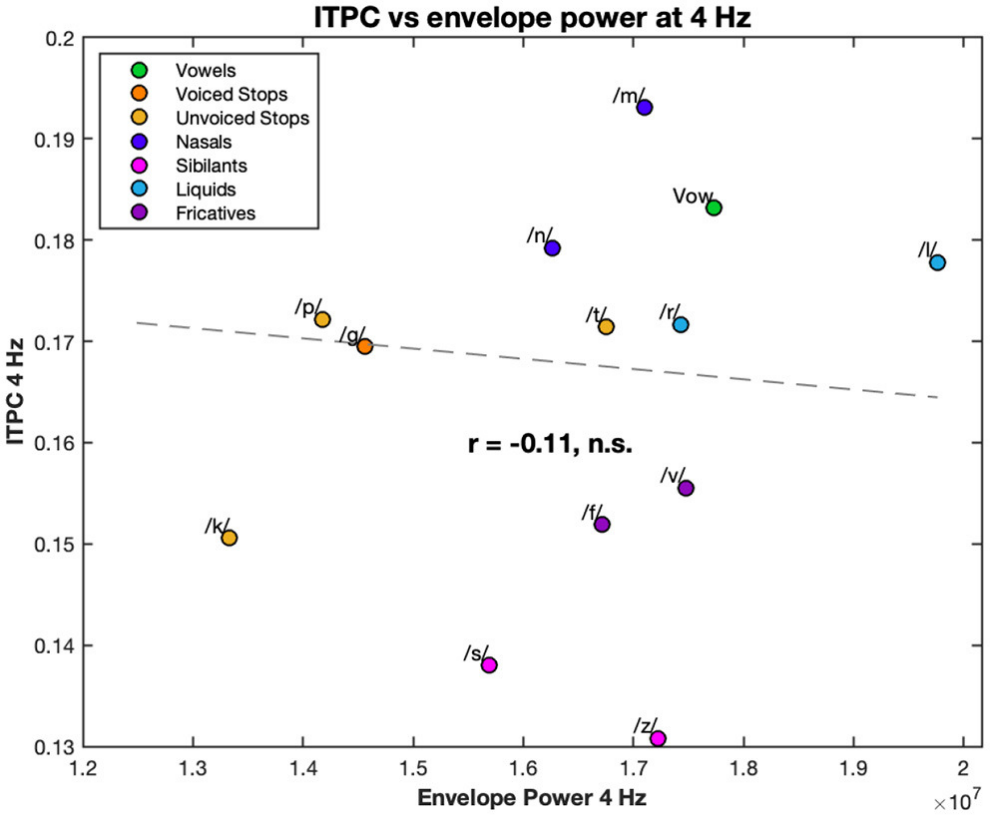
Correlation between the 4 Hz ITPC and the 4 Hz power of the stimulus envelopes. The EEG ITPC at 4 Hz is plotted against the power of the envelope of each stream. Both are averaged over condition. Each color corresponds to a different condition (see legend). Dashed line represents the line of best fit (least means squares). The correlation coefficient is given below this line. n.s., not significant.

Lastly, we calculated the correlation coefficient between the ITPC at 4 Hz and at the harmonic frequencies, and the power of the stream envelopes at the same frequencies. None of these correlations were significant (see, for example, the correlation between the ITPC at 4 Hz and power of the stimulus envelope at 4 Hz in Figure 7).

## 4. Discussion

Here, we have seen that differences in the syllable-initial consonants led to differences in neural phase locking to streams of near-isochronous CV syllables. Using a near-isochronous stimulus produced peaks in the ITPC at 4 Hz; harmonic responses were also seen in the ITPC, at 8, 12 and 16 Hz. Harmonic peaks are a natural consequence of a periodic stimulus (Zhou et al., 2016) and, here, these were investigated in further analyses. We found that the ITPC responses to the different stimulus conditions at 4 Hz and at its harmonics were moderately correlated with each other. It was convenient to decorrelate these using PCA. The first principal component, ITPC1, showed significant differences between different phonemic groups.

The relationship between neural entrainment and features of the acoustic envelopes was studied. A wide range of different measures were considered, with the measures that describe the “sharpness” of the initial rise in the envelope showing the strongest relationship to neural entrainment. As with the ITPC, PCA was used to calculate a combined measure of edge markers, with this first principal component and ITPC1 showing a correlation coefficient of 0.84. This strong relationship was not a mere (by-)product of the PCA analysis, and was also observed when, for example, the ITPC at 4 Hz and the latency to the MD (the maximum derivative of the envelope) were compared directly (Figure 6).

As early studies have suggested (Heil, 1997, 2003), we found that it is features of the first derivative of the envelope that mostly trigger neural activity to sound stimuli, in this case speech. Both the size and latency of the MD have each been previously suggested as the most important landmark in neural phase locking to speech (Oganian and Chang, 2019). It has been proposed that the MD corresponds to the CV transition within syllables, as this encodes the highest “rate of change” (Oganian and Chang, 2019; Kojima et al., 2021). However, the MA has also been suggested as a primary landmark for neural entrainment to speech because this contains the highest acoustic energy of the syllable (Ghitza, 2013) and indeed, speech intelligibility was affected more by masking vowel information from the syllabic nucleus with noise, but not consonant information from either the onset or offset of syllables (Fogerty and Kewley-Port, 2009; Fogerty et al., 2010). A more recent experiment by Kojima et al. (2021) showed that responses to continuous speech were only reliably evoked by the peak in the positive derivative, but not the MA, even if these neural responses were also closely aligned in time with the peaks of syllable envelopes. It is possible that syllable-segregation differs between subjects and might vary in response to different frequency profiles for hearing acuity: while we only included participants who reported having unimpaired hearing it is possible that some participants had undetected frequency-specific hearing loss. It would be interesting to perform an experiment with a design similar to the one reported here but to include a hearing assessment.

Our study only used CV syllables, not the wider variety of syllable structure which is encountered in continuous speech. Acoustic edge features might look quite different in more complex syllables, including consonant clusters or those in which a liquid is present in the coda; we would hope that our approach, with different stimuli, would be useful in quantifying this. Similarly, we have only included speech sounds in this study; the segregation of speech into syllables is important for comprehension and it seems likely that the phenomena we have observed here depend on features that are typical of speech sounds such as phonemes. Conversely, though, this early stage of processing may not be specialized to phonemic categorization rather than to a broader class of “speech-like” sounds which share acoustic features, like edges, with speech. It would be interesting to test this using a similar experiment with non-speech sounds. It is also interesting to compare the brain responses we have measured, as in for example Figure 3, to the differences between consonants, as measured by confusion between them, see Goldstein (1980), Miller and Nicely (1955), and Shepard (1972): there are notable similarities and it will be interesting in future experiments to combine EEG recording and perceptual tasks to explore this more formally.

Here, our focus was on the relationship between the initial syllabic phonemes and neural responses. We measured how different phoneme features affect neural entrainment to speech. It is known that different consonants are processed by discrete regions of the superior temporal gyrus (STG) of the auditory cortex, and an ECoG study showed that separate electrode clusters responded to phonemes differing in their manner of articulation and voicing (Mesgarani et al., 2014). However, an fMRI study also showed that there is substantial overlap between regions responsible for the processing of different consonant groups (Arsenault and Buchsbaum, 2015). Furthermore, the STG, which is the area of the brain showing discrete processing of phonemes, is also responsible for envelope encoding (Hamilton et al., 2020) and comprehension (Binder et al., 2000). Our results, which show that different syllable-initial consonants lead to differences in speech chunking, complement the fact that there is a functional and anatomical overlap between the processing of phonemes and that of the speech envelope. Thus, phoneme encoding may not entirely be subordinate to syllabic tracking and the processing of phonemes at syllable onset may, as suggested in Di Liberto and Lalor (2017), form part of the mechanism which supports the tracking of syllables. We have not examined the responses on a region-by-region or electrode-by-electrode basis—this is because our focus here was on the overall response to phoneme features and averaging over all electrodes removes electrode choice as a source of ambiguity; it would be useful to look in future at the variation across electrodes and to embed the analysis in a full Bayesian model.

Interestingly, as in, for example, Figure 7, none of the ITPC values, at 4 Hz or harmonics, were correlated with the power of the stream envelopes at the corresponding frequencies, suggesting that phonemic landmarks rather than the overall stimulus shape play the larger role in neural entrainment. Furthermore, this provides clear evidence of the non-linear relationship between stimulus and response; something that is also evident from the strength harmonics at multiples of the 4 Hz frequency. One potential confound to this, and similar, experiments is an artifact from the electromagnetic dynamics of the headphones. However, the absence of correlation in the power and the presence of harmonics are indicative a biological origin. Nonetheless, the potential headphone artifacts must be acknowledged and it would be useful in future experiments to rule them out.

Our study was limited in a number of other ways: it used near-isochronous, meaningless stimuli rather than natural speech, consisting of only CV syllables with a restricted set of consonants and vowels. It would be interesting to examine more natural English stimuli and to extend it to other languages with different syllable patterns. Our goal was to extract a robust response by using a frequency-tagged paradigm with multiple repetitions of the same stream; it would be worthwhile to consider other approaches, such as the one described in Di Liberto et al. (2015), which allows the investigation of natural speech.

A potentially useful methodological outcome of our experiment concerns the use of near-isochronous stimuli. As described in the Methods, the syllables in our streams were not exactly 250 ms long. Nonetheless, reliable ITPC peaks at 4 Hz and at harmonic frequencies were found with no evidence of 'bleeding' of power into the bins adjacent to the 4 Hz bin. Most generally it supports the suggestion in Ghitza (2011) that the theta oscillators track a quasi-periodic input rhythm to aid speech comprehension, while suggesting that the oscillators described in that work exploit edge-features of the input to synchronize with speech. Testing a far broader range of stimuli is needed to verify the limits.

The results reported here reflect neural responses averaged over the entire scalp. Most phonemic-acoustic information has been found to be processed by the STG. Replicating this study using technology, such as MEG, with better spatial resolution would be useful in showing to what extent these results are specific to speech-encoding brain areas. A modeling approach would also be useful in revealing the underlying neural phenomenon behind similar results.

The present research has potential applications in understanding dyslexia. It has been found that children with dyslexia have difficulty processing the rising amplitudes of the envelopes, across different languages (Thomson et al., 2013). Some researchers claim that envelope rise times are associated with phonemic spectro-temporal information (Tallal, 2004), while others suggest that they help convey stress patterns (Goswami and Leong, 2013). Indeed, dyslexic children seem to benefit from both phonemic and stress-training procedures: Thomson et al. (2013) describe how both a phonemic intervention procedure, in which children matched the sounds of different syllables to a target one, and a rhythm identification procedure, in which they repeated the stress of certain words using non-word syllables such as “dee” (for example, where the stress in the words "Harry Potter" was illustrated by “DEEdee DEEdee”) have beneficial effects on reading and writing. This study showed that the rhythm intervention had an advantage over the phonemic one in terms of the children's envelope rise time discrimination. It would be interesting to see if interventions tackling envelope rise time perception in relation to syllabic landmarks would also improve reading skills.

In conclusion, this study showed, for the first time, that neural entrainment to speech depends on the features of the speech segments (in the present case, the manner of articulation or voicing of the phonemes) located at the onsets of syllables. These features may also differ in the acoustic edges they provide, which we have quantified in various ways. We found that the most important landmark for neural entrainment to consonant-vowel syllables was the latency to the maximum derivative of the envelope—which supports previous findings—but its magnitude did not seem to matter.

## Data Availability Statement

The original contributions presented in the study are publicly available. This data can be found here: The audio files for the stimuli, as well as the raw and processed EEG datasets from this study, can be found on the Open Science Framework website, under the project name “CV syllables and neural entrainment to speech”, https://osf.io/3c6tv/. The code used for running and analyzing the experiment is available in the GitHub repository, https://github.com/phonemes-and-speech-entrainment/cv_syllables.

## Ethics Statement

The studies involving human participants were reviewed and approved by University of Bristol Human Participants Ethics Committee. The patients/participants provided their written informed consent to participate in this study.

## Author Contributions

MC ran the experiments, performed data analysis, and wrote the initial draft of the paper. All authors contributed to the design of the experiment and editing of the paper.

## Funding

MC and experimental costs were supported by the Wellcome Trust Doctoral Training Programme in Neural Dynamics at Bristol University, grant no. 105207/Z/14/Z. NK acknowledges the support from the International Laboratory for Social Neurobiology of the Institute for Cognitive Neuroscience HSE, RF Government grant # 075-15-2019-1930.

## Conflict of Interest

The authors declare that the research was conducted in the absence of any commercial or financial relationships that could be construed as a potential conflict of interest.

## Publisher's Note

All claims expressed in this article are solely those of the authors and do not necessarily represent those of their affiliated organizations, or those of the publisher, the editors and the reviewers. Any product that may be evaluated in this article, or claim that may be made by its manufacturer, is not guaranteed or endorsed by the publisher.
