# Supplementary material for: Syllable-Initial Phonemes Affect Neural Entrainment to Consonant-Vowel Syllables
Source: Front Neurosci. 2022 Jun 14;16:826105. doi: 10.3389/fnins.2022.826105 (PMC9237462; doi:10.3389/fnins.2022.826105)
Supplement: Supplementary file 1 [file Data_Sheet_1.PDF]

Supplementary Material

STIMULUS AND APPARATUS

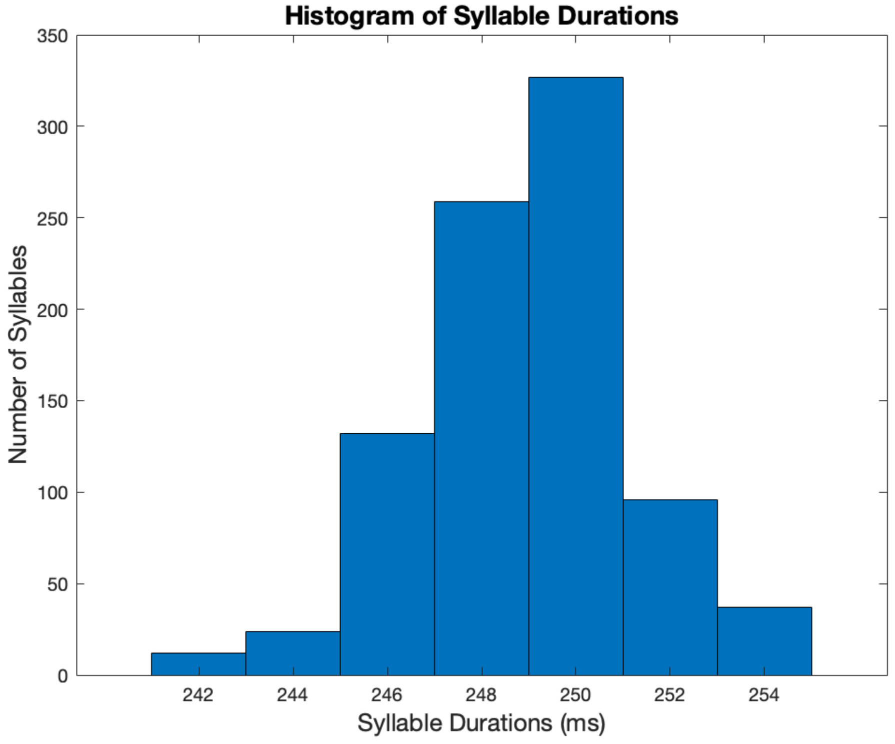

Figure S1. Syllable durations. The stimulus was nearly-isochronous: this is a histogram of syllable durations.

EVOKED POWER

The main analysis was performed using ITPC, however the evoked power

$$P(f) = \left| \frac{\sum_{k=1}^{K} X_k(f)}{K} \right|^2 \tag{S1}$$

was also calculated; $X_k(f)$ is the value of the Fourier transform at frequency f.

In the evoked power, the characteristic alpha response was seen between 8-12 Hz, and only the peak at syllabic rate was significantly higher than activity at nearby frequencies (Maximum $p < .01$, FDR-corrected), as can be seen in Fig.S4.

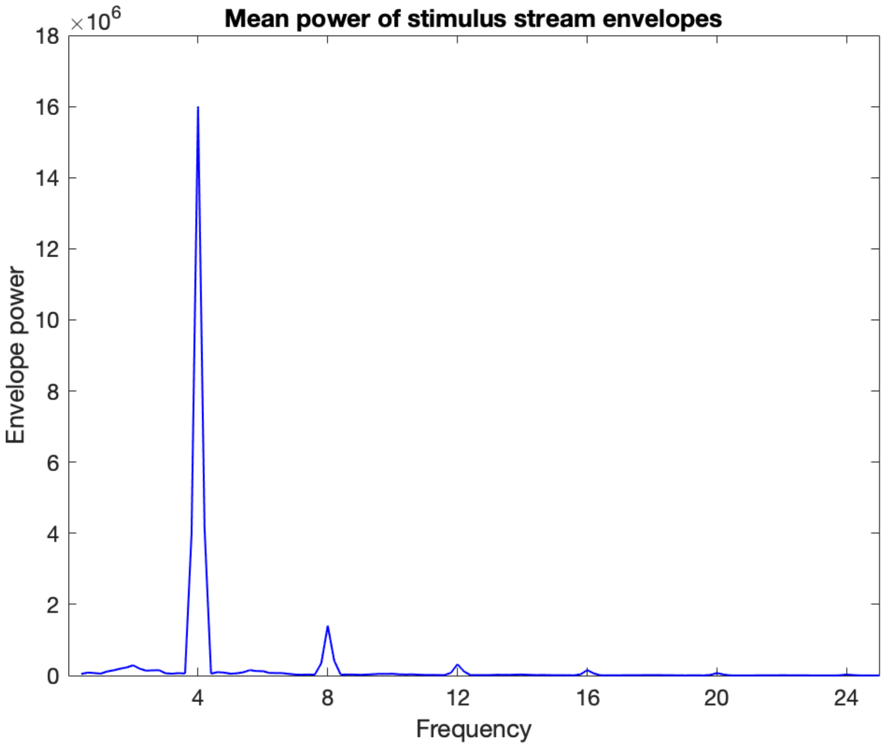

Figure S2. Power of stimulus envelopes. The average power of all stimulus envelopes (calculated for the 10 s streams) is plotted as a function of frequency. Note that this shows peaks at 4 Hz and harmonics.

CONSONANT GROUP STATISTICAL COMPARISONS

Repeated measures ANOVAS to test differences between the 15 conditions were conducted fo the 4 Hz evoked power: $F_{14,24} = 2.53$, $p = .002$.

In terms of ITPC, we also conducted repeated-measures ANOVAS and Bonferroni-corrected T-tests on the five (sibilants, nasals, fricatives, liquids, stops) and three consonant (sibilants/fricatives, nasals/liquids, stops) groups. The results from the five-group and three-group ANOVAs were significant for the ITPC at 4 Hz: these were $F_{4,24} = 7.90$, $p < .001$ and $F_{2,24} = 9.60$, $p < .001$, respectively. Only the five-group ANOVA was significant for the ITPC results at the 8 Hz harmonic: $F_{4,24} = 6.37$, $p < .001$.

The 4 Hz evoked power showed some results which were similar to findings from the 4 Hz ITPC. The five and three-group repeated measures ANOVAs were significant: $F_{4,24} = 4.62$, $p = .002$ and $F_{2,24} = 4.91$, $p = .011$, respectively. Bonferroni-corrected T-tests showed that in the five-group comparisons, only sibilants showed significantly less power values than stops ($p = .046$), and in the three-group comparisons, sibilants/fricatives also showed less 4 Hz evoked power than stops ($p = .018$).

The significant uncorrected p-values for pairwise comparisons were, as follows: for ITPC1 (five groups) - sibilants ¡ fricatives, $p = .025$; sibilants ¡ liquids, $p = .002$; sibilants ¡ nasals, $p < .001$; sibilants ¡ stops, $p < .001$; fricatives ¡ nasals, $p = .002$; fricatives ¡ stops, $p = .012$; liquids ¡ nasals, $p = .006$; for ITPC1 (three groups) - sibilants/fricatives ¡ nasals, $p < .001$; sibilant/fricatives ¡ stops, $p < .001$; for ITPC2 (five groups) - liquids ¡ nasals, $p = .002$; for the evoked power at 4 Hz (five groups) - sibilants ¡ liquids, $p = .041$; sibilants ¡ nasals, $p = .009$; sibilants ¡ stops, $p = .004$; fricatives ¡ nasals, $p = .026$; and three groups - sibilants/fricatives ¡ nasals/liquids, $p = .025$; sibilants/fricatives ¡ stops, $p = .006$.

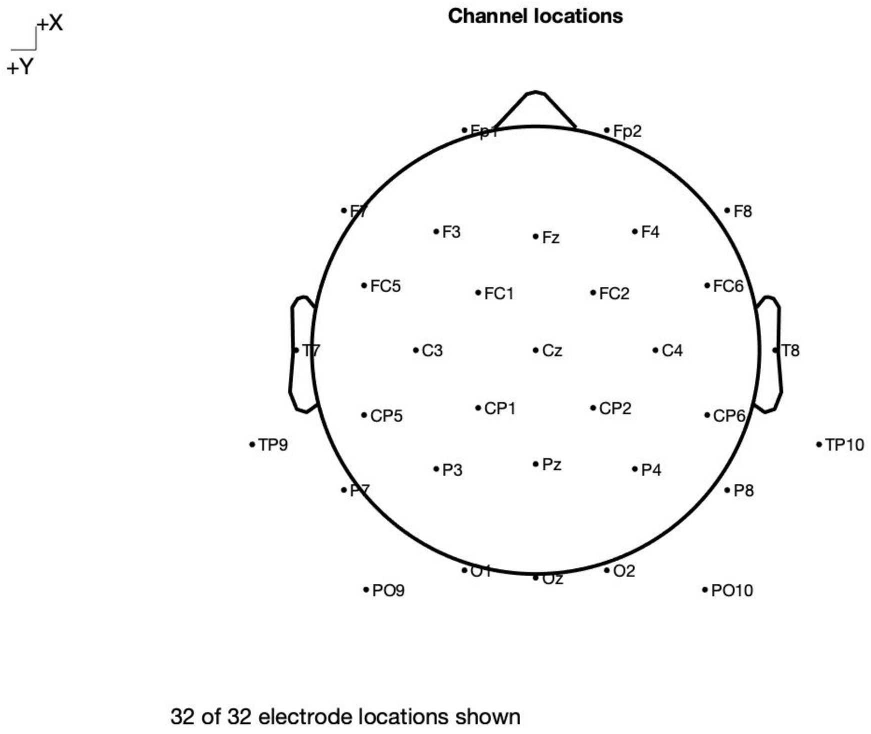

Figure S3. Electrode location for the apparatus used. EEG channel names and scalp configuration (read top to bottom, left to right). Plotted using EEGLAB.

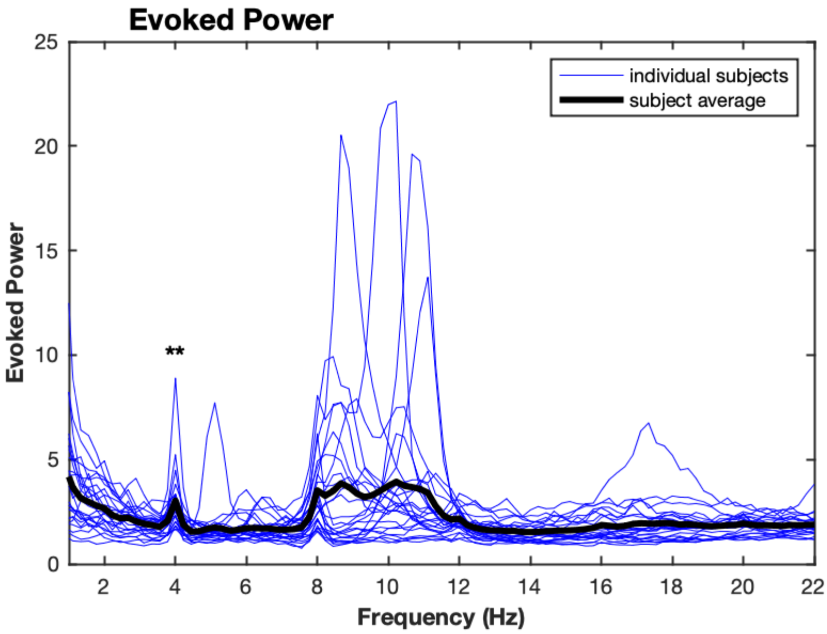

Figure S4. Evoked power.The evoked power averaged over channels and conditions is plotted as a function of frequency, for individual subjects as well as a subject average. Only the peak in evoked power at 4 Hz is significantly different from values at its neighboring frequency bins. ** $p < .01$.

EDGE MARKER CORRELATIONS

	MD	Time MD	Sharpness	Gini	MA	Time MA	Plateau
MD	1.0000	-.1201	.6988***	.2758*	.4859***	-.3637**	-.0209
Time MD	-.1201	1.0000	-.4172***	.6824***	.0150	.6802***	-.4145***
Sharpness	.6988***	-.4172***	1.0000	-.0435	.4802***	-.7295***	-.1292
Gini	.2758*	.6824***	-.0435	1.0000	.4266***	.4002***	-.5288***
MA	.4859***	.0150	.4802***	.4266***	1.0000	-.0438	-.3680***
Time MA	-.3637**	.6802***	-.7295***	.4002***	-.0438	1.0000	-.0790
Plateau	-.0209	-.4145***	-.1292	-.5288***	-.3680	-.0790	1.0000

Table S1. Edge-marker correlation coefficients. MA = maximum amplitude of the envelope; MD = maximum derivative of the envelope; *p¡.05, **p¡.01, ***p¡.001.

ITPC AND EDGE-MARKER CORRELATION COEFFICIENTS

Lastly, we also calculated correlation coefficients between the ITPC at 4 Hz, as well as its harmonic values at 8, 12 and 16 Hz, and each of the edge markers used in the analyses. These can be seen in Table S2. Like ITPC1, the ITPC at 4 Hz showed significant relationships with the MD latency ($r = .91$, $p < .001$), sharpness ($r = .55$, $p = .032$), the Gini index ($r = .71$, $p = .003$), the MA latency ($r = -.78$, $p < .001$) as well as 80% of the MA latency ($r = -.81$, $p < .001$). These showed the same trends as ITPC1 but the correlations were stronger. Moreover, the positive correlation between the ITPC at 4 Hz and the syllable plateau was significant ($r = .57$, $p = .030$), as opposed to the one with ITPC1, which was only marginally significant.

The ITPC at 8 Hz only showed significant negative relationships with the MD latency ($r = -.57$, $p = .003$) and 80% of the MA latency ($r = -.52$, $p = .048$). The ITPC at 12 Hz was the only harmonic of the ITPC to show significant positive relationships with the values of the MD ($r = .80$, $p < .001$) and MA ($r = .55$, $p = .034$), as well as sharpness ($r = .71$, $p = .003$). The 12 Hz ITPC was also negatively correlated with the MA latency ($r = -.58$, $p = .024$) and 80% of its value ($r = -.53$, $p = .044$).The ITPC at 16 Hz did not show any significant relationships with the edge markers.

Edge Markers	4 Hz		8 Hz		12 Hz		16 Hz
Sharpness	.55	*	.50		.71	***	.08
MA	.23		.32		.55	*	-.44
MA Latency	-.78	***	-.51		-.58	*	-.14
80% of MA Latency	-.81	***	-.52	*	-.53	*	-.23
Plateau	.57	*	.34		-.12		.48
Gini Index	-.71	**	-.36		.17		-.37
MD	.23		.44		.80	***	-.10
MD Latency	-.91	***	-.57	*	-.21		-.25

Table S2. Correlation coefficients between ITPC at 4 Hz and its harmonics and the values of edge markers. Stars represent significance values: * $p < .05$, ** $p < .01$, *** $p < .001$. No stars indicate the correlation coefficient was not significant

PRINCIPAL COMPONENT ANALYSIS

We performed a principal component analysis (PCA) on the first four ITPC peaks, as the 16 Hz peak did not show strong correlations with the other ones and further peaks got smaller and noisier. The PCA was performed on a 45 (stimulus stream) x 25 (participants) x 4 (peaks) matrix, resized to a 1125 x 4 matrix, so individual scores for the 25 participants were kept after the PCA. Each principal component was also a 45 x 25 matrix: we averaged across the 3 streams in each condition (phoneme) to obtain the final 15 x 25 matrices on which further statistics were conducted (i.e, condition averages based on voicing and manner of articulation).

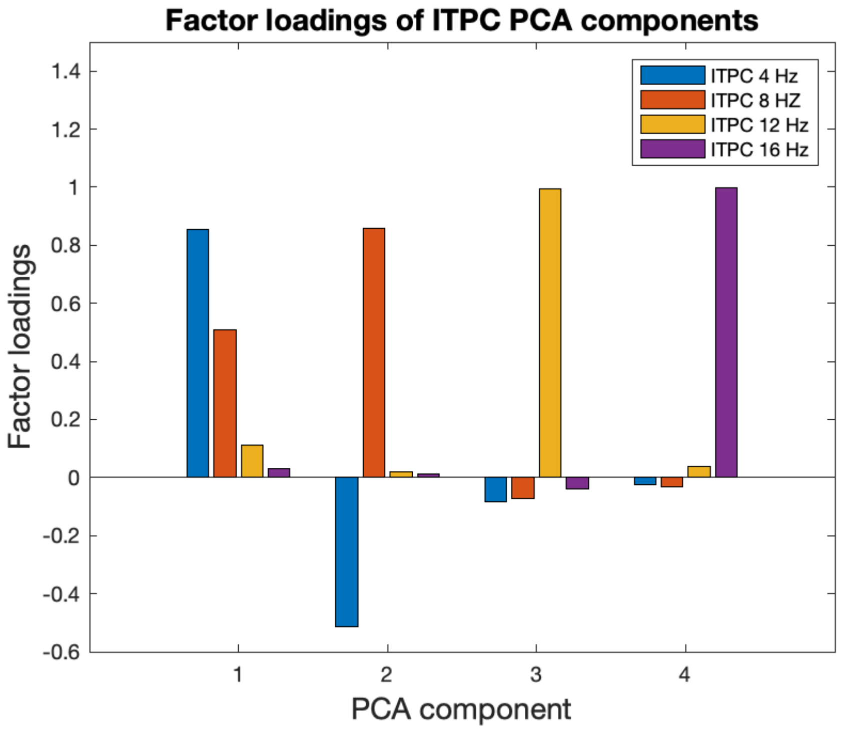

Figure S5. PCA factor loadings for the four principal components of ITPC. PCA calculated for the ITPC at 4, 8, 12 and 16 Hz led to four components in which each of the ITPC values contributed with different factor loadings. The graph illustrates the factor loadings for each peak in ITPC, at each component.

We also performed PCA on the five edge markers which showed most significant correlations with the ITPC peaks (and between each other). The matrix that went into this PCA was of size 75 (syllable) x 5 (edge). The principal components were vectors of length 75 and we averaged across syllables (5) in each condition, so the final vectors to be correlated with the ITPC principal components had 15 points.

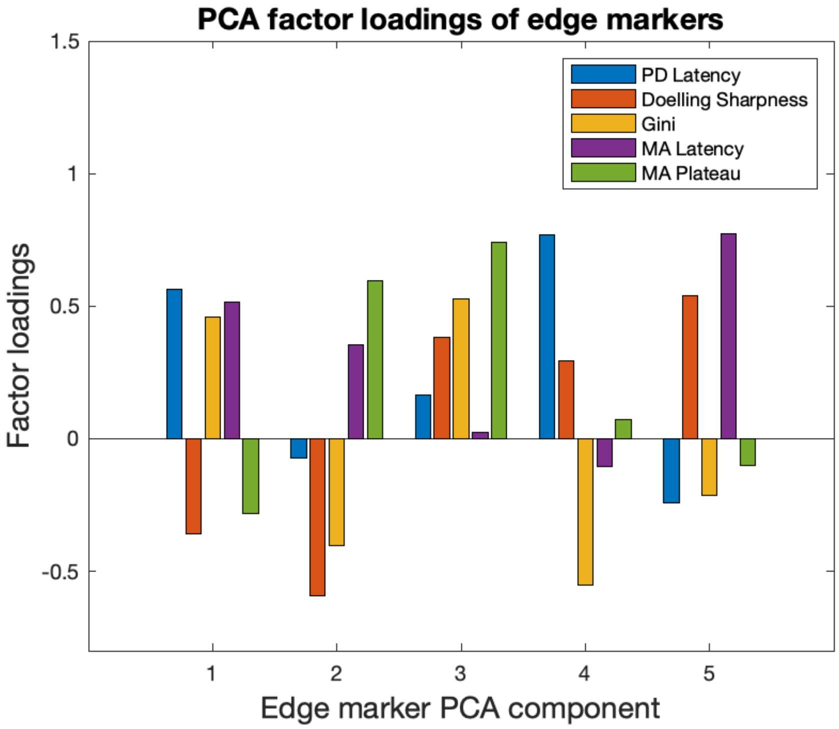

Figure S6. PCA factor loadings for the four principal components of the edge markers. PCA calculated for the edge markers led to five components in which each of the edge markers contributed with different factor loadings. The graph illustrates the factor loadings for each edge marker.

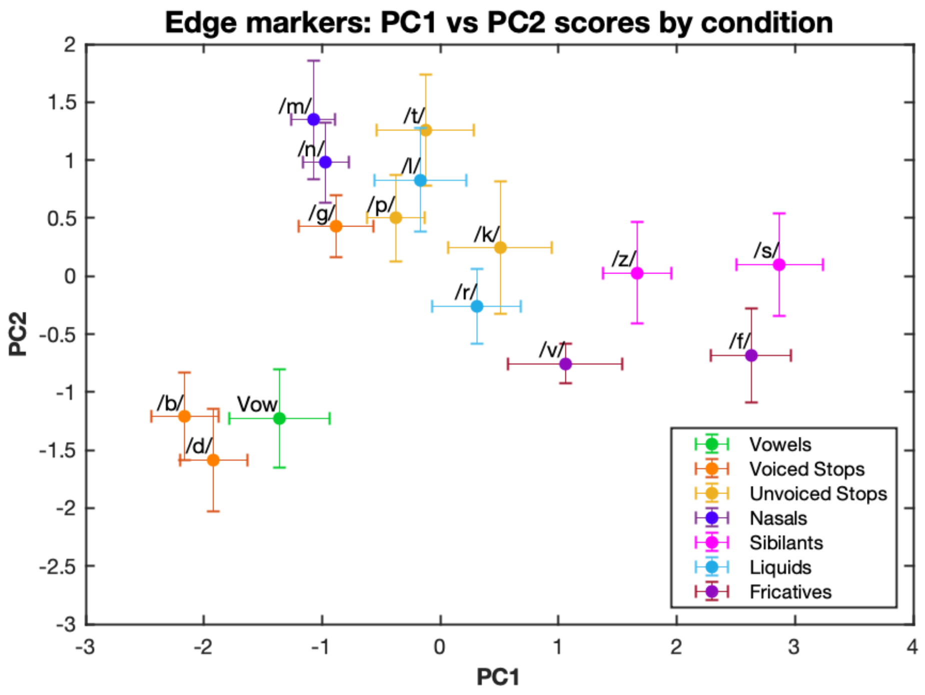

Figure S7. First and second principal components of the edge markers. Conducting PCA on the five edge markers (latencies of MD and MA, sharpness, syllable plateau and the Gini index) of each syllable resulted in different component scores for each of the syllables. The scores of PC1 and PC2 were averaged over syllables pertaining to their corresponding conditions. Colors represent the same phonemic groups as in Fig. 3.
